# Growing microglia in the lab

**DOI:** 10.7554/eLife.103535

**Published:** 2024-10-28

**Authors:** Jonathan M Levenson, Hio Tong Kam, Dong Feng Chen

**Affiliations:** 1 FireCyte Therapeutics, Inc Boston United States; 2 Schepens Eye Research Institute of Mass. Eye and Ear, Department of Ophthalmology, Harvard Medical School Boston United States

**Keywords:** human iPSC, Microglia, PLX-5622, retinal transplantation, RPE, NaIO3, Mouse

## Abstract

Transplanting microglia derived from human stem cells into mice reveals new possibilities for treating neurodegenerative eye diseases.

**Related research article** Ma W, Zhao L, Xu B, Fariss RN, Redmond TM, Zou J, Wong WT, Li W. 2024. Human iPSC-derived microglial cells integrated into mouse retina and recapitulated features of endogenous microglia. *eLife* **12**:RP90695. doi: 10.7554/eLife.90695.

As the global population gets older, age-related diseases are becoming more prevalent, with many linked to dysfunctional immune responses. In the central nervous system, microglia are resident immune cells that maintain homeostasis, respond to injury and regulate inflammation. However, these cells can become dysregulated over time, and the resulting inflammation has been implicated in age-related neurodegenerative diseases, including Alzheimer’s disease (Cai et al., 2022) and Parkinson’s disease (Tansey et al., 2022), as well as diseases that affect the retina, such as glaucoma (Wei et al., 2019; Pan et al., 2023). However, despite extensive research into methods for shifting microglia from a dysfunctional state back into a state that can protect the nervous system, there are currently no drugs that have been approved to do this.

Microglia derived from induced pluripotent stem cells (iPSCs) – adult cells that have been reprogrammed to have the same properties as embryonic stem cells – offer a powerful way to study human microglia in healthy and diseased states. When sourced directly from patients, these cells offer a renewable, patient-specific model with the potential to be used therapeutically to replace dysfunctional microglia with healthier versions without the risk of immune rejection. Now, in eLife, Wai T Wong (Tiresias Bio), Wei Li (National Eye Institute) and colleagues – including Wenxin Ma as first author – report the results of initial efforts to develop iPSC-derived microglia that can be used to treat retinal diseases (Ma et al., 2024).

First, Ma et al. optimized previously reported methods for the production of human microglial cells from iPSCs. This enabled them to continuously produce microglia at a purity (often greater than 95%) and a scale that could support both research and the commercialization of their approach. Testing the method in five distinct stem cell lines showed that it was broadly applicable, increasing the likelihood that it could be used to generate microglia from individual patients.

Gene expression profiles from the resulting microglia closely resembled those of native microglia and were clearly distinct from the progenitor iPSCs. Challenging the iPSC-derived microglia with bacteria revealed that they displayed two hallmark functional activities of native microglia: (i) they secreted proteins associated with inflammatory responses when exposed to bacterial toxins; (ii) they engulfed particles coated with bacteria.

Next, the team optimized a procedure for replacing dysfunctional microglia in the retina with healthy microglia derived from iPSCs. In mice, native microglia in the retina were depleted by inhibiting a receptor required for their survival, and fluorescent human iPSC-derived microglia were then injected into the subretinal space. Over the course of 3–6 months, the human microglial cells successfully repopulated the retina and integrated across all retinal layers (Figure 1). Some native mouse microglia were also present in the retina, but at greatly reduced numbers. The human microglia assumed characteristic shapes that indicated they had taken on a protective and supportive functional state.

**Figure 1.**
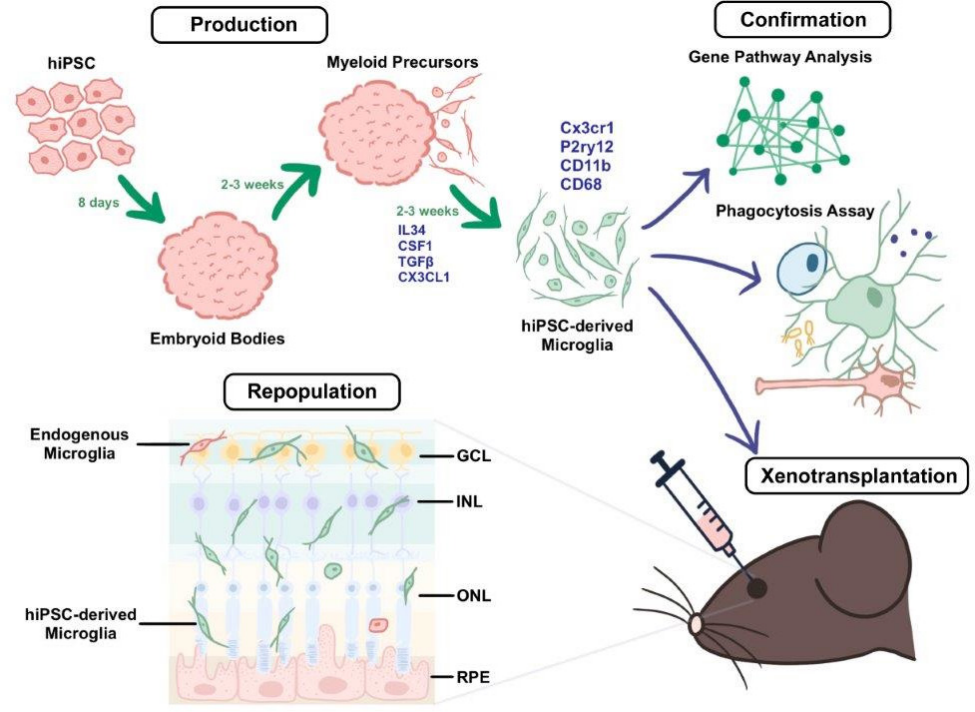
A method for producing human iPSC-derived microglia and transplanting them into mice. During the production stage (top left), embryoid bodies derived from human iPSCs (induced pluripotent stem cells; pink) develop into myeloid precursors, which then differentiate into microglia (green cells) after exposure to a cocktail of cytokines (IL-34, CSF1, TGFβ, and Cx3cl1). The microglia derived from the iPSCs express typical microglia markers (such as Cx3cr1, P2ry12, CD11b, and CD68). During the confirmation stage (top right), the properties of the microglia are validated through a combination of gene profiling, comparative Gene Pathway Analysis, phagocytosis assays (which probe the ability of the microglia to engulf particles coated with bacterial proteins), and xenotransplantation (which involves transplanting the microglia into the subretinal space of adult mice; bottom right). Xenotransplantation is followed by the repopulation stage (bottom left), in which the microglia derived from the iPSCs become integrated and distributed across the different retinal layers in a pattern consistent with endogenous microglia (red cell). GCL: ganglion cell layer; INL: inner nuclear layer; ONL: outer nuclear layer; RPE: retinal pigment epithelium.

Lastly, Ma et al. determined whether the transplanted human microglia could respond to retinal damage. Killing photoreceptors (the cells that detect light) by administering high doses of the chemical sodium iodate (Kannan and Hinton, 2014), caused the human microglia to transiently proliferate and migrate into the layer of the retina containing the photoreceptors. Moreover, the human microglia began to consume the cellular debris created by the dying photoreceptors. These responses mirror the actions of native microglia and reflect the critical ability of the iPSC-derived microglia to protect the remaining healthy photoreceptor cells.

With this innovative approach, a biopsy of skin cells from a patient with glaucoma could be transformed into the very cells that might halt or prevent blindness. Even more intriguing, the mouse model developed by Ma et al. to allow transplantation of iPSC-derived human microglia represents a valuable platform for discovering even more therapies for retinal diseases. Taken together, the findings of Ma et al. represent a significant step forward in developing new therapeutic approaches for treating age-related central nervous system disorders where microglial dysfunction plays a central role.

## References

[bib1] Cai Y, Liu J, Wang B, Sun M, Yang H (2022). Microglia in the neuroinflammatory pathogenesis of Alzheimer’s disease and related therapeutic targets. Frontiers in Immunology.

[bib2] Kannan R, Hinton DR (2014). Sodium iodate induced retinal degeneration: new insights from an old model. Neural Regeneration Research.

[bib3] Ma W, Zhao L, Xu B, Fariss RN, Redmond TM, Zou J, Wong WT, Li W (2024). Human iPSC-derived microglial cells integrated into mouse retina and recapitulated features of endogenous microglia. eLife.

[bib4] Pan L, Cho K-S, Wei X, Xu F, Lennikov A, Hu G, Tang J, Guo S, Chen J, Kriukov E, Kyle R, Elzaridi F, Jiang S, Dromel PA, Young M, Baranov P, Do C-W, Williams RW, Chen J, Lu L, Chen DF (2023). IGFBPL1 is a master driver of microglia homeostasis and resolution of neuroinflammation in glaucoma and brain tauopathy. Cell Reports.

[bib5] Tansey MG, Wallings RL, Houser MC, Herrick MK, Keating CE, Joers V (2022). Inflammation and immune dysfunction in Parkinson disease. Nature Reviews Immunology.

[bib6] Wei X, Cho K-S, Thee EF, Jager MJ, Chen DF (2019). Neuroinflammation and microglia in glaucoma: time for a paradigm shift. Journal of Neuroscience Research.

